# Annealing Effect on Structural, Optical and Electrophysical Properties of ZnSe Nanocrystals Synthesized into SiO_2_/Si Ion Track Template

**DOI:** 10.3390/ma17164149

**Published:** 2024-08-22

**Authors:** Aiman Akylbekova, Alma Dauletbekova, Zein Baimukhanov, Liudmila A. Vlasukova, Abay Usseinov, Nuray Saduova, Abdirash T. Akilbekov, Vladimir A. Pankratov, Anatoli I. Popov

**Affiliations:** 1 Faculty of Physics and Technology, L.N. Gumilyov Eurasian National University, Astana 010000, Kazakhstan; alma_dauletbek@mail.ru (A.D.); zeinb77@mail.ru (Z.B.); usseinov_ab@enu.kz (A.U.); samuraikas21@mail.ru (N.S.); akilbekov_at@enu.kz (A.T.A.); 2 Faculty of Radiophysics and Computer Technologies, Belarusian State University, Kurchatova Street 5, 220045 Minsk, Belarus; vlasukova@mail.ru; 3 Institute of Solid State Physics, University of Latvia, 8 Kengaraga, LV-1063 Riga, Latvia; popov@latnet.lv; 4 Institute of Physics, University of Tartu, W. Ostwald Str. 1, 50411 Tartu, Estonia

**Keywords:** SiO_2_/Si track template, chemical deposition, ZnSe nanocrystals, quantum chemical calculation, thermal annealing, PL in UV-VIS spectral range

## Abstract

We report the results of synthesis of zinc selenide (ZnSe) nanocrystals into SiO_2_/Si track templates formed by irradiation with 200 MeV Xe ions up to a fluence of 10^7^ ions/cm^2^. Zinc selenide nanocrystals were obtained by chemical deposition from the alkaline aqueous solution. Scanning electron microscopy, X-ray diffractometry, Raman and photoluminescence spectroscopy, and electrical measurements were used for characterization of synthesized ZnSe/SiO_2nanoporous_/Si nanocomposites. XRD data for as-deposited precipitates revealed the formation of ZnSe nanocrystals with cubic crystal structure, spatial syngony F-43m (216). According to non-empirical calculations using GGA-PBE and HSE06 functionals, ZnSe crystal is a direct-zone crystal with a minimum bandgap width of 2.36 eV and anisotropic electronic distribution. It was found that a thermal treatment of synthesized nanocomposites at 800 °C results in an increase in ZnSe nanocrystallites size as well as an increase in emission intensity of created precipitates in a broad UV-VIS spectra range. However, vacuum conditions of annealing still do not completely prevent the oxidation of zinc selenide, and a formation of hexagonal ZnO phase is registered in the annealed samples. The current–voltage characteristics of the synthesized nanocomposites proved to have n-type conductivity, as well as increased conductivity after annealing.

## 1. Introduction

Nowadays, semiconductor nanomaterials are of great interest for R&D due to their new and superior properties compared to well-known and thoroughly studied ones for bulk materials [1]. Among them is ZnSe, a II-VI semiconductor with a direct bandgap of 2.7 eV, whose excellent optoelectronic and electrical properties in the nanostructured state have attracted much attention in recent years [1,2,3,4,5,6]. The luminescent light emission of different ZnSe nanostructures can vary in a wide range from blue to red depending on synthesis techniques and post-growth annealing regimes [5,6,7,8]. The enhanced luminescence properties of ZnSe nanostructures make them suitable for preparing more efficient blue and green LEDs and lasers for medical applications [9,10,11,12]. To date, a variety of different nanostructured ZnSe forms (nanowires, nanobelts, nanocrystals, nanoporous material, etc.) has been synthesized [1,4,6,7,8,9,10,11,13,14,15]. Nanostructuring the surface of ZnSe and other semiconductor crystals and the corresponding processes have been studied in detail in [16,17,18,19,20].

In order to obtain new promising materials for optoelectronics, nanoelectronics, and sensors, a detailed study of nanomaterials based on zinc-based selenides and complex oxides synthesized in SiO_2_/Si templates is also of undoubted interest. So-called ion track template synthesis is one of the simplest and most inexpensive methods for obtaining metal and semiconductor nanoclusters and nanowires [21,22]. In our papers [21,22,23,24,25,26,27,28], methods of ZnO, ZnSe_2_O_5_, ZnSeO_3_, CdTe, and CdO nanocrystal deposition into SiO_2_/Si templates were successfully developed.

It is known that ZnSe nanostructures as well as polycrystalline ZnSe obtained by the CVD technique at elevated temperatures (800–1000 °C) have revealed good crystalline quality and excellent optoelectronic properties [29,30].

Another approach to improve the crystallinity and decrease the defect level in nanostructured materials is a thermal annealing, which is considered as an efficient tool to tune the properties of semiconductor nanomaterials. In the case of ZnSe nanostructures, generally, annealing is carried out in air (see, for example, [31,32]). However, ZnSe high-temperature processing in air may instigate oxide phases in the nanocrystals, which is a major issue. In order to prevent oxidation and maintain synthesized material’s quality, annealing in vacuum conditions could be undertaken. In accordance with [30], in the case of vacuum annealing, the oxidation of ZnSe crystals by residual oxygen becomes noticeable at a temperature higher than 1150 K (880 °C).

In our experiment, the next step after the synthesis of nanocrystalline zinc selenide in the SiO_2_/Si templates was to carry out high-temperature treatment at a temperature of 800 °C in an attempt to improve their crystallinity and optical properties.

Therefore, the aim of the present work is to investigate the annealing effect and the optical and electrophysical properties of ZnSe/SiO_2_/Si nanocomposites formed by template synthesis. We also performed ab initio calculations to confirm our findings and improve our understanding of how synthesis conditions affect the physical properties of nanocrystals. We report the results of characterization of nanocrystals deposited into a nanoporous silica layer of SiO_2_/Si track template before and after annealing, using X-ray diffraction, micro-Raman, and photoluminescence spectroscopy. We believe that these results are important for controlling the synthesis process of nanostructured semiconductor materials in terms of their optoelectronic properties and applications in the field of photoelectronic devices.

## 2. Materials and Methods

### 2.1. Formation of SiO_2_/Si Track Templates

**Figure 1:**
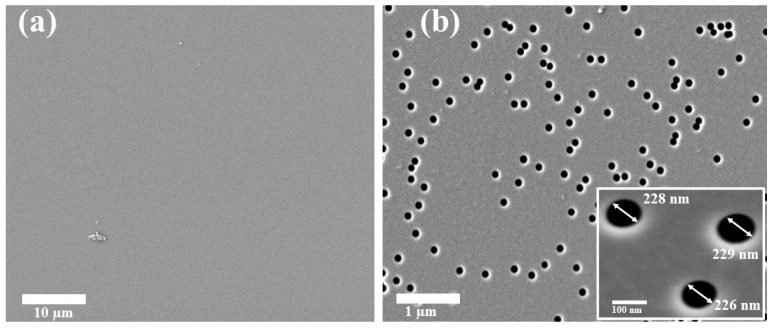
SEM images of the surface of a-SiO_2_/Si-n track template (**a**) after irradiation; (**b**) after etching for 10 min.

The initial a-SiO_2_/Si-n structure was obtained by thermal oxidation of n-type Si substrate (100) with a diameter of 100 mm in a wet oxygen atmosphere at 900 °C. According to ellipsometry data, the thickness of the oxide layer was 700 nm. The oxidized substrate was irradiated with Xe ions (200 MeV, 10^7^ ions/cm^2^), and the samples of 5 × 5 mm and 10 × 10 mm were cut from the irradiated substrate. These irradiations were successfully carried out at the DC-60 heavy ion accelerator located at the Institute of Nuclear Physics, Kazakhstan, which in recent years has proven to be a powerful experimental facility for performing such experiments [23,24,25,26,27,28,33,34,35,36,37]. Before track etching, the surface of the samples was cleaned with isopropanol for 15 min in a 6.SB25-12DTS ultrasonic cleaner. To form nanopores at the site of latent ion tracks in SiO_2_ film, the chemical etching of the samples was carried out at a room temperature in a 4% aqueous solution of hydrofluoric acid (HF) with addition of palladium chloride (PdCl_2_). PdCl_2_ concentration in the etchant was 0.05 g L^−1^. After etching, the created templates were washed in deionized water (18.2 MOhm) and dried in air. Figure 1 depicts SEM images of the irradiated SiO_2_/Si sample before and after track etching.

One can see that the chemical treatment results in a formation of clearly visible conical pores with practically the same diameters (for this sample nanopore’s diameters ranged from 226 to 229 nm). The etched track depth and diameter can be tuned via etchant concentration and etching time.

### 2.2. Synthesis of ZnSe by Chemical Precipitation Method

The initial aqueous solution for ZnSe precipitation included zinc chloride (ZnCl_2_), hydrazine hydrate (H_6_N_2_O), ammonia (NH_3_·H_2_O), and sodium hydroxide (NaOH). Sodium selenosulfate (Na_2_SeSO_3_) was used as a source of selenium for ZnSe synthesis. In order to prepare sodium selenosulfate, 5 g of Se, and 30 g of Na_2_SO_3_ were added to 200 mL of distilled water, stirred, put into an oil bath at 90℃, and purged with argon for 24 h.

**Figure 2:**
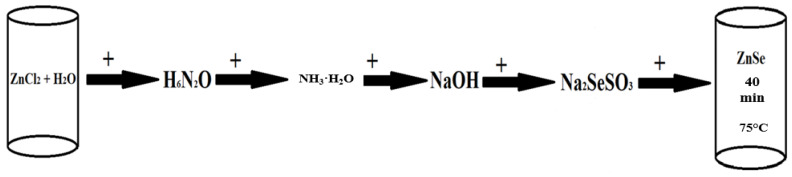
Schematic illustration of ZnSe NC synthesis.

For ZnSe precipitation, a solution was prepared, containing 6.75 g ZnCl_2_ and 2 mL of H_6_N_2_O per 100 mL of distilled water, under constant stirring. pH = 11 was reached with the addition of 25 drops of ammonia. Subsequently, the solution temperature was slowly increased (3 °C/min), and when the desired temperature of 75 °C was reached, 45 mL of freshly prepared Na_2_SeSO_3_ was added under constant stirring. The process of ZnSe deposition into SiO_2_/Si templates was carried out at a solution temperature of 75 °C; the duration of deposition was 40 min. The deposited samples were washed with distilled water and dried in air. Figure 2 shows the scheme of synthesis of ZnSe nanoclusters (NCs).

### 2.3. Diagnostics of SiO_2_/Si Templates with Deposited Nanoprecipitates

The morphology of template surfaces and nanoprecipitates were analyzed using the scanning electron microscope (SEM) JSM 7500F. The crystallographic structure of the precipitates was investigated by X-ray diffraction (XRD). XRD patterns were obtained using a Rigaku SmartLab X-ray diffractometer (XRD). The elemental analysis of synthesized precipitates was carried out using energy-dispersive X-ray spectroscopy (EDX) on a scanning electron microscopy Hitachi TM3030. Raman spectra were recorded at room temperature using the Spectrum Raman spectrometer (NT-MDT). A 473 nm solid-state laser was used as the excitation source. A spectral resolution of 1 cm^−1^ was provided by an 1800/500 diffraction grating. The laser was focused on the sample to a spot of 2 μm diameter using a 100X objective. The signal accumulation time was 100–200 s. Photoluminescence (PL) spectra were recorded using the CM2203 spectrofluorometer. Radiation by Xe lamp with a wavelength of 300 nm was used for PL excitation.

**Figure 3:**
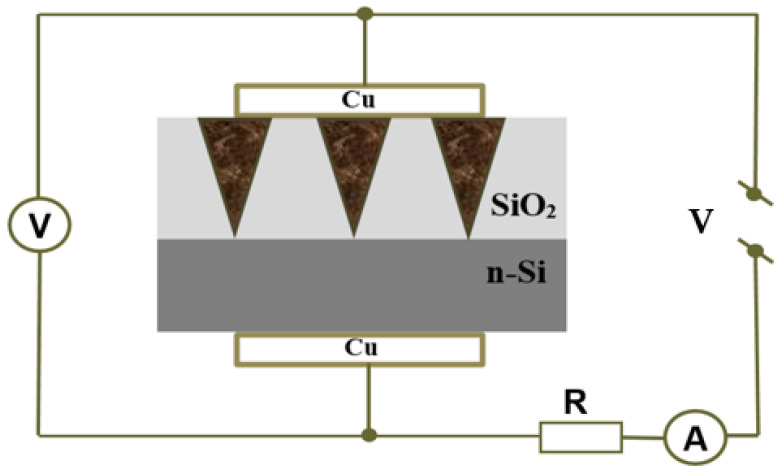
The scheme of the setup for current–voltage characteristic measurements.

To study the electrical properties of arrays of synthesized nanowires, a current source HP 66312A and multimeter 34401A from “Ajilent” (USA) were used. The current–voltage characteristics (CVCs) were taken from an array of filled nanochannels, with an area of 0.3 cm^2^. The scheme of the setup is shown in Figure 3. Copper electrodes were deposited via thermal evaporation to ensure the contacts’ ohmage, repeatability of results and mechanical stability. The CVCs were measured in constant voltage mode from −6 to 6 V in steps of 0.5 V. All CVCs were plotted using a second-order polynomial fit.

In order to investigate the annealing effect on the created ZnSe/SiO_2_/Si nanocomposites, a heat treatment using an AVERON furnace at 800 °C in vacuum for 60 min was carried out.

## 3. Results

**Figure 4:**
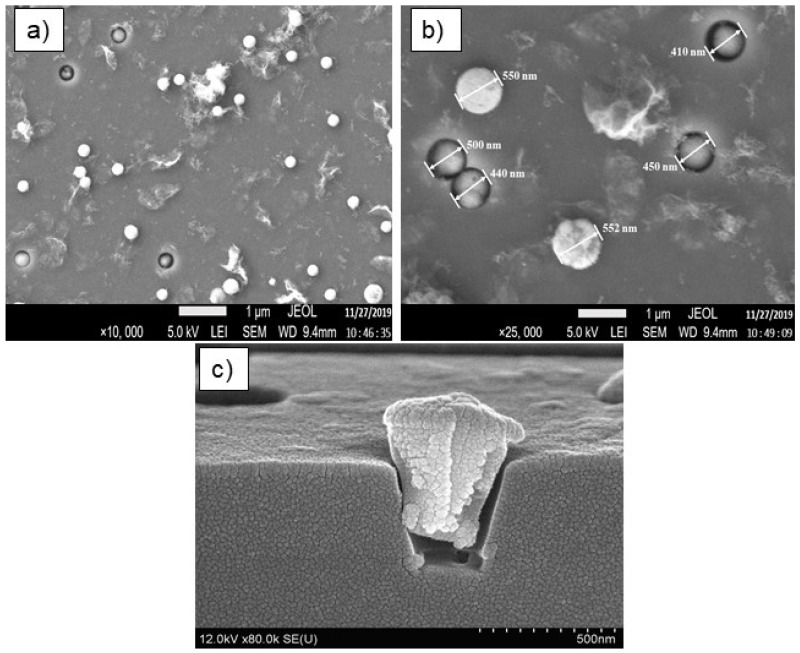
SEM surface images after chemical precipitation of ZnSe at 75 °C for 40 min (**a**,**b**), and (**c**)—cross-section of a pore filled with precipitates.

Figure 4 shows the surfaces of the templates after the ZnSe chemical precipitation at 75 °C for 40 min.

One can see that precipitation for 40 min results in a fulfilment of etched pores in silica layer. However, there are some not entirely filled pores (the pores with diameters 410–550 nm) as well as ones with “caps” at the SiO_2_ surface, as shown in Figure 4. These “caps” result in an increase in pores diameter up to 552 nm.

**Figure 5:**
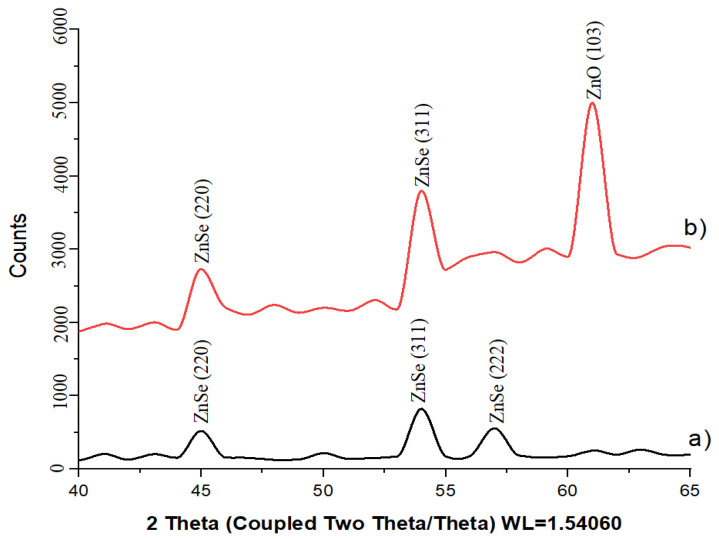
X-ray diffractograms of the deposited nanocrystals before and after annealing: (**a**) as-deposited sample; (**b**) annealed sample.

Figure 5 depicts X-ray diffractograms of the as-deposited and annealed samples.

The corresponding crystallographic parameters of nanoprecipitates calculated from XRD data are summarized in Table 1. Analysis of XRD data for samples after deposition proves the formation of ZnSe nanocrystals with cubic crystal structure, spatial syngony F-43m (216). In our experiment the reflections from the planes with Miller indices (220), (311), (222) are observed. The calculated lattice parameters of zinc selenide agree well with the literature data [31,38,39].

One can see from Figure 5 and Table 1 that a vacuum annealing at 800 °C still does not completely prevent the oxidation of zinc selenide. The X-ray diffraction pattern of the annealed sample reveals the formation of a ZnO phase with a hexagonal structure (spatial syngony P63mc (186), JCPDS 75-0576). Reflections from the plane with Miller indices (103) are observed, confirmed by the presence of a single peak at 2θ = 61.95°. An appearance of two extra bands in the range of (55–60) 2θ is additionally observed at the XRD of the annealed sample. These bands may indicate the formation of byproducts of oxidation like ZnSeO_3_ or ZnSeO_4_. It should be noted that annealing results in and increase in ZnSe nanocrystal size.

**Figure 6:**
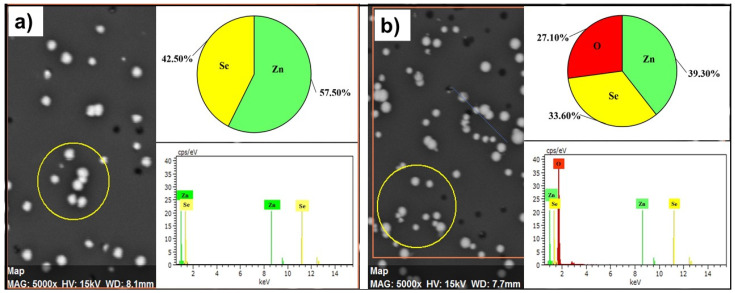
EDX spectra of the as-deposited and annealed precipitates (**a**,**b**).

Figure 6 presents the EDX spectra of the as-deposited and annealed nanocrystals. EDX data prove the XRD results concerning partial oxidation of ZnSe nanocrystals after annealing. The elemental composition of as-deposited nanocrystals calculated from EDX data corresponds to Zn (42.5 at. %) and Se (57.5 at. %). After annealing the nanocrystal’s composition corresponds to Zn (39.3 at. %) Se (33.6 at. %) O (27.3).

### 3.1. Raman Spectroscopy

**Figure 7:**
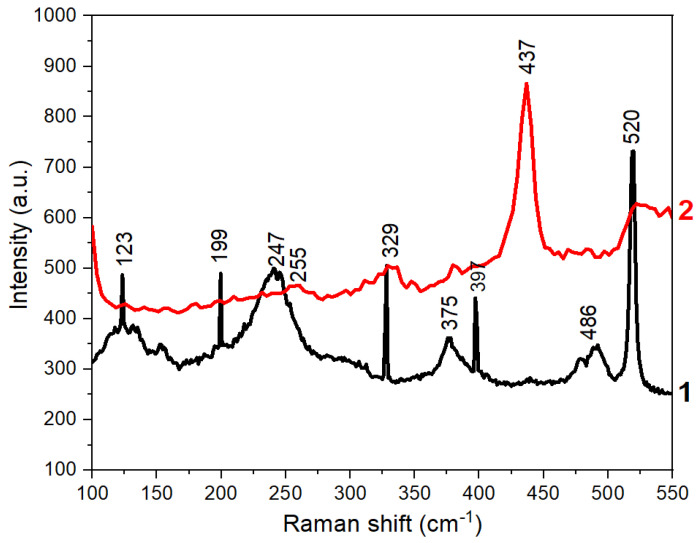
Raman spectra of deposited precipitates before and after annealing at 800 °C. Curves 1 and 2 in the spectra correspond to the as-deposited and annealed samples correspondingly.

Figure 7 shows the Raman spectra of the synthesized nanoparticles before and after annealing. The Raman spectrum of the as-deposited sample depicts five asymmetric bands at 123, 199, 247, 375, and 486 cm^−1^. The peaks at 247 and 486 cm^−1^ are assigned to the longitudinal optical phonon modes of ZnSe as 1LO and 2LO, respectively [41]. A shoulder at 211 cm^−1^ on the band at 247 cm^−1^ is also observed which is assigned to the transverse optical (TO) phonon mode. The band located at about 123 cm^−1^ possibly results from the interfaces [42]; the intensive band at 520 cm^−1^ can be ascribed to the signal from Si wafer. We could not identify the band at 375 cm^−1^. One could suggest that it can be attributed to the transverse optical (TO) phonon mode.

According to Refs. [41,42,43,44], the LO phonon frequencies of monocrystalline films and bulk species of ZnSe at room temperature correspond to 254 cm^−1^ and 255 cm^−1^, respectively. For polycrystalline ZnSe nanoparticles, the bands of TO and LO phonons were observed at 210 cm^−1^ and 255 cm^−1^, correspondingly, and gave a broad RS band due to the high surface area-to-volume ratio of small particles.

Compared to these results, the LO and TO phonon’s bands of ZnSe nanoparticles synthesized in our experiment are shifted towards lower frequencies, which may be due to the effect of small size and large surface area of the synthesized nanoparticles. There is also a rather intensive broad band corresponding to the LO phonon first overtone, at 498–501 cm^−1^, indicating a weak anharmonicity [45].

The Raman spectrum of precipitates annealed at 800 °C shows asymmetric bands at 253 and 329 cm^−1^ and an intensive peak at 437 cm^−1^. The bands at 253 and 329 cm^−1^ are assigned to the longitudinal optical phonon modes of ZnSe as 1LO and 2LO, respectively. The peak at 437 cm^−1^ can be assigned to E1 high vibrational mode of ZnO [46]. An appearance of the band of ZnO in the RS spectrum is additional proof of the partial oxidation of ZnSe nanocrystals during high-temperature processing.

### 3.2. Photoluminescence

**Figure 8:**
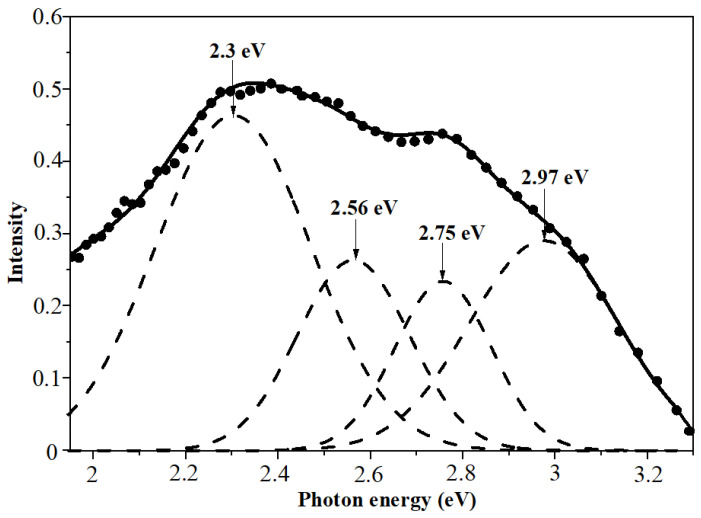
Differential PL spectra of as-deposited ZnSe nanocrystals with Gaussian curves.

**Figure 9:**
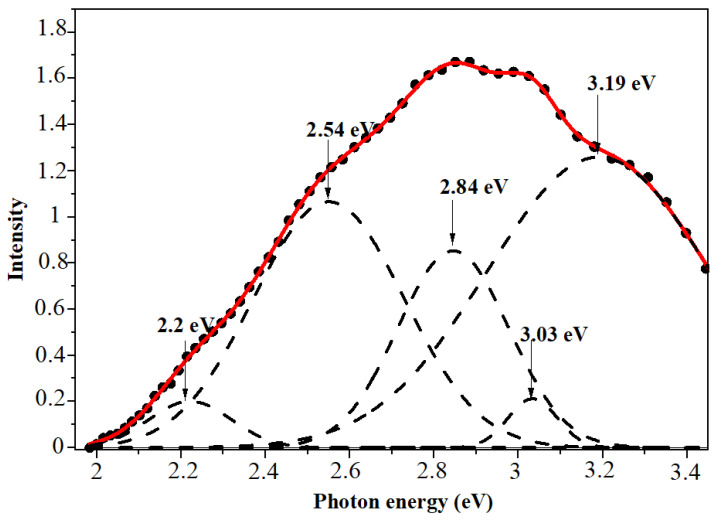
Differential PL spectra of the annealed ZnSe nanocrystals.

PL spectroscopy of as-deposited and annealed precipitates reveals a light emission in a broad UV-VIS range of spectra. Figure 8 and Figure 9 depict the PL spectra of the investigated nanoparticles. One can see that the luminescence spectra are quite complex components and can be deconvoluted into five Gaussian curves. As can be seen from Figure 8, the PL spectrum of the as-deposited ZnSe composes of bands at 1.93 eV, 2.3 eV, 2.56 eV, 2.75 eV, and 2.97 eV. The emission observed at about 1.9 eV and 2.3 eV can be ascribed to Se and Zn vacancies, respectively [46]. The band at 2.56 eV (484 nm) is possibly due to some donor–acceptor pairs associated with Zn/Se vacancies and intermediate states [47]. The band at 2.75 eV corresponds to Zn interstitials [48]. The luminescence band located at 2.97 eV, close to the absorption edge, can be assigned to the band-to-band transition in ZnSe crystallites. This value, which is higher than the corresponding value of room temperature band gap energy for ZnSe bulk crystal, clearly indicates the increase in the band gap of the ZnSe nanocrystallites.

As a result of annealing in air, part of the zinc selenide structure is oxidized, turning into zinc oxide. This in turn has a strong effect on the resulting structure of the photoluminescence spectra. If the photoluminescence peaks are attributed to electron transitions on ZnSe defects before annealing, then after annealing, the spectrum shifts to the right (blue shifting) and new high-energy peaks appear, which are already associated with donor–acceptor defects of the ZnO lattice. As is known, ZnO has a larger forbidden zone compared to ZnSe (3.4 vs. 2.7 eV) [49]. According to similar photoluminescence spectra of ZnO, a donor–-acceptor pair transition was observed at 3.217 eV, with phonon replicas at 3.145, 3.073, and 3.001 eV [50], which is very close to the observed 3.03 and 3.19 eV peaks. Moreover, after annealing at 800 °C, the line intensity increases, which is associated with an increase in the number of electron transitions due to the addition of zinc oxide luminescence.

### 3.3. Electrophysical Properties

**Figure 10:**
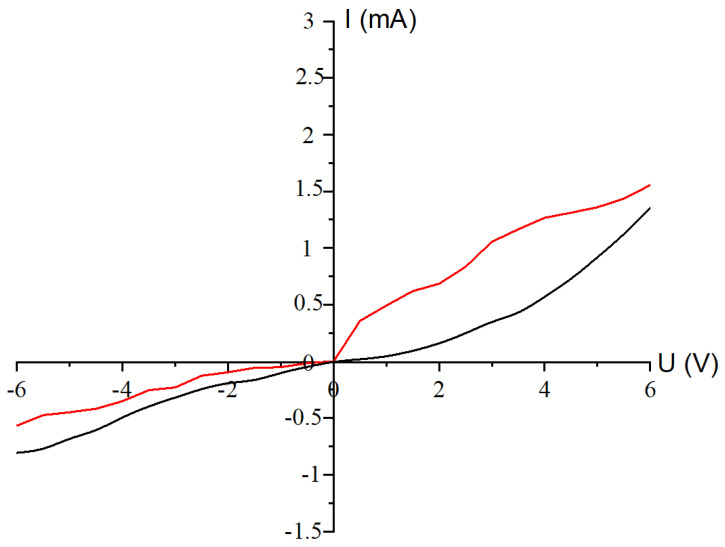
Current–voltage characteristic of precipitates annealed at 800 °C: black line—before annealing; red line—after annealing. The ZnSe deposition time is 40 min.

Figure 10 depicts the current–voltage characteristics of the synthesized zinc selenide structures before and after annealing in forward and reverse directions, taken at room temperature.

The CVC measurements of the ZnSe structure after annealing revealed an increase in electrical conductivity after annealing. This effect may be due to changes in the crystal structure (improved crystallinity and grain size increase), reduction in intergranular boundaries (grain boundary domains), and removal of some impurities. An increase in electronic conductivity with increasing annealing temperature can be the explained by growth in crystallite sizes, and the decrease in the width of the forbidden zone is shown in [51].

According to the CVCs, the obtained nanocrystal samples are characterized with pronounced electronic conductivity, which is due to the presence of defects in the crystal lattice. Two variants of the origin of conductivity can be assumed: (i) the conductivity is due to the presence of intrinsic defects (such as selenium vacancy or zinc interstitial), or (ii) the presence of hydrogen-containing impurities (such as hydrogen or water). For example, intrinsic defects in oxide semiconductors (ZnO, SnO_2_, and In_2_O_3_) were considered as the source of the pronounced electrical conductivity [52]. However, later, high-precision calculations showed the impurity nature of the electronic conductivity [53].

### 3.4. Ab Initio Calculations

We also performed non-empirical calculations of the ZnSe crystal in the terms of the density functional theory using the hybrid Heyd–Scuseria–Ernzerhof range-separated functional (HSE06) [54] and compared the calculated parameters with experimental data.

The calculated values of lattice parameters, crystallographic cell volume, and density of the crystal are presented in Table 1. As a result, the hybrid HSE06 functional gives better agreement with experiment data than other known calculations using a standard GGA or LDA functional [52,55,56]. Such a hybrid functional allows for us to perform very accurate calculations of the band gap, unlike the standard LDA- or GGA-type functional. Indeed, the relative underestimation of the band gap is 12% (2.36 vs. 2.7 eV), which is consistent on average with the accuracy of hybrid DFT methods. The pure DFT-GGA gives a band gap of 1.19 eV.

**Figure 11:**
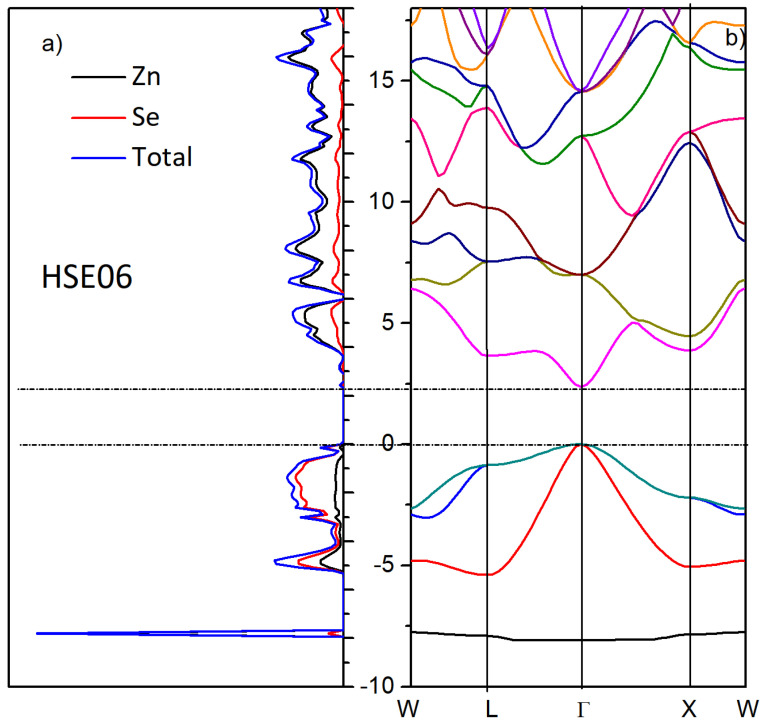
Density of states (**a**) and zone structure (**b**) of cubic ZnSe (HSE06). Horizontal lines denote the boundaries of the valence band and conduction band.

The electronic structure along the highly symmetric k-points of the Brillouin zone is shown in Figure 11. We determined that the crystal has a direct (Г-Г) gap with fairly pronounced anisotropy of the valence and conduction bands, which indicates a low effective electron mass. Indeed, as reported previously, the electron effective mass in ZnSe m_eff_ = 0.17m_0_ (see [57,58]). Analysis of the density of states showed that the top of the valence band is presented by s-p states of Se atoms, and the bottom of the conduction band consists of d-states of Zn atoms. The Mulliken atomic charges evidence typical ionic compounds with charge distribution on the zinc and selenium atoms +0.76/−0.76 e.

This charge distribution is characteristic of ionic crystals to which ZnSe belongs. The electronic structure as a function of the wave vector direction is shown in Figure 11b. As can be seen, the crystal is direct-zoned with a minimum bandgap width of 2.36 eV, and the electronic distribution is anisotropic. According to previously published works, the experimental values of Eg band gap energy are in the region of 2.663 [59], 2.67 [60,61], 2.688 [62], 2.69 [63], and 2.692 eV [64]. Finally, it is important to note all the most important exciton parameters—such as electron and hole effective masses, zero-frequency exciton polarizability, exciton binding energy, and ground-state energy of direct excitons in ZnSe—have been studied in detail and given in [65].

## 4. Conclusions

Zinc selenide nanocrystals were successfully synthesized in SiO_2_/Si track template by chemical deposition in a water–alkali medium from of ZnCl_2_ and Na_2_SeSO_3_ as Zn and Se precursors at 75 °C. Track template was formed by irradiation with 200 MeV Xe ions up to fluence of 10^7^ ions/cm^2^ followed by chemical etching of latent tracks in an aqueous solution of hydrofluoric acid.

Analysis of XRD diffractograms for as-deposited precipitates revealed the formation of ZnSe nanocrystals with cubic crystal structure, spatial syngony F-43m (216). The formation of the ZnSe crystalline phase was proven by RS spectroscopy, too. Non-empirical calculations of the ZnSe crystal performed in the terms of the DFT using GGA-PBE and HSE06 functionals proved a direct-zone ZnSe crystal with a minimum bandgap width of 2.36 eV and anisotropic electronic distribution.

A thermal treatment of synthesized nanocomposites at 800 °C in vacuum resulted in a formation of a hexagonal ZnO phase due to the oxidation of deposited ZnSe precipitates by residual gases (O_2_ and H_2_O) in a vacuum environment as well as in an increase in ZnSe nanocrystallite size.

For as-deposited and annealed precipitates, an emission in a broad UV-VIS range of spectra was observed, and its intensity increased after annealing. It was supposed that this emission arose presumably due to zinc and selenium vacancies as well as Zn interstitials and emission at the ZnSe and ZnO band gap edges. The current–voltage characteristics of the synthesized nanocomposites proved to have n-type conductivity as well as an increase of conductivity after annealing. An increase in PL intensity and electrical conductivity of the template with deposited nanocrystals after annealing gives reason to believe that heat treatment is an effective way to enhance these parameters in the SiO_2nanoporous_/Si template with deposited ZnSe nanocrystals for possible application as an active material in white light-emitting diodes (LEDs).

## Figures and Tables

**Table 1:** ZnSe crystallographic characteristics data.

Property	Our Results			Other
	Before annealing	After annealing	Calculated by DFT (HSE06)	Continenza et al. [40]
Type of structure	Cubic	Cubic + hexagonal ZnO	–	Cubic
Space group	216 (F-43m)	216 (F-43m), 186 (P63mc)	–	216 (F-43m)
L, nm	25	25.7; 32.3	–	–
Phase content, %	100	78.8; 21.2	–	–
a, Å	5.59	5.63; a = 3.23, c = 5.20	5.71	5.668
V, Å^3^	174.87	174.88; 49.27	186.25	–
*ρ*, g/cm^3^	5.48	5.36; 5.65	5.13	5.27

## Data Availability

The raw data supporting the conclusions of this article will be made available by the authors on request.
